# Protective Effects of Glutamine and Leucine Supplementation on Sepsis-Induced Skeletal Muscle Injuries

**DOI:** 10.3390/ijms222313003

**Published:** 2021-11-30

**Authors:** Yu-Chen Hou, Man-Hui Pai, Jin-Ming Wu, Po-Jen Yang, Po-Chu Lee, Kuen-Yuan Chen, Sung-Ling Yeh, Ming-Tsan Lin

**Affiliations:** 1 Master Program in Food Safety, College of Nutrition, Taipei Medical University, Taipei 11031, Taiwan; ychou@tmu.edu.tw; 2 School of Food Safety, College of Nutrition, Taipei Medical University, Taipei 11031, Taiwan; 3 Nutrition Research Center, Taipei Medical University Hospital, Taipei 11031, Taiwan; 4 Department of Anatomy and Cell Biology, School of Medicine, College of Medicine, Taipei Medical University, Taipei 11031, Taiwan; pai0507@tmu.edu.tw; 5 Department of Surgery, National Taiwan University Hospital and College of Medicine, National Taiwan University, Taipei 11031, Taiwan; kptkptkpt@yahoo.com.tw (J.-M.W.); paulpjyang@gmail.com (P.-J.Y.); d97421103@ntu.edu.tw (P.-C.L.); dtsurg51@gmail.com (K.-Y.C.); sangling@tmu.edu.tw (S.-L.Y.); 6 Department of Surgery, National Taiwan University Hospital Hsin-Chu Branch, Hsinchu County 302, Taiwan

**Keywords:** monocyte, macrophage, calpain, hypoxia-inducible factor-1α, mitochondria

## Abstract

This study investigated the effects of l-glutamine (Gln) and/or l-leucine (Leu) administration on sepsis-induced skeletal muscle injuries. C57BL/6J mice were subjected to cecal ligation and puncture to induce polymicrobial sepsis and then given an intraperitoneal injection of Gln, Leu, or Gln plus Leu beginning at 1 h after the operation with re-injections every 24 h. All mice were sacrificed on either day 1 or day 4 after the operation. Blood and muscles were collected for analysis of inflammation and oxidative damage-related biomolecules. Results indicated that both Gln and Leu supplementation alleviated sepsis-induced skeletal muscle damage by reducing monocyte infiltration, calpain activity, and mRNA expression levels of inflammatory cytokines and hypoxia-inducible factor-1α. Furthermore, septic mice treated with Gln had higher percentages of blood anti-inflammatory monocytes and muscle M2 macrophages, whereas Leu treatment enhanced the muscle expressions of mitochondrion-related genes. However, there were no synergistic effects when Gln and Leu were simultaneously administered. These findings suggest that both Gln and Leu had prominent abilities to attenuate inflammation and degradation of skeletal muscles in the early and/or late phases of sepsis. Moreover, Gln promoted the switch of leukocytes toward an anti-inflammatory phenotype, while Leu treatment maintained muscle bioenergetic function.

## 1. Introduction

Sepsis is systemic inflammation induced by severe infection that commonly occurs in critically ill patients. Prolonged weakness is a major cause of morbidity and mortality in this critical illness [1,2], and sepsis is a major risk factor for acquired muscle weakness [3,4]. Abundant evidence indicates that sepsis induces myopathies characterized by increased proteolytic degradation and decreased protein synthesis, which may lead to impaired muscle function and a poor quality of life. There are multiple mechanisms contributing to the development of myopathies in critical illnesses [5]. Among these, excessive localized elaboration of proinflammatory cytokines, free-radical generation, activation of proteolytic pathways, and altered mitochondrial bioenergetic function play critical roles in sepsis-associated myopathic changes [6]. Inhibition of these pathways may attenuate muscle injuries and prevent the progression of sepsis-induced myopathies.

Skeletal muscles, the main producer of glutamine in the body, are responsible for maintaining glutamine levels in catabolic conditions, which is usually accompanied by muscle depletion [7]. l-glutamine (Gln) is a pharmaconutrient that has immunomodulatory, anti-inflammatory, and antioxidative properties in critical illnesses [8,9]. A previous study indicated that Gln supplementation inhibits whole-body protein degradation in patients with muscular dystrophy [10] and attenuates muscular inflammation which contributes to muscle regeneration in diabetic mice subjected to limb ischemia [11]. Leucine, an essential amino acid, is considered a modulator of skeletal muscle protein metabolism by inhibiting protein breakdown and stimulating protein synthesis [12]. However, the efficacy of standalone l-leucine (Leu) interventions in preventing muscle wasting under disease conditions is doubtful and inconclusive [13]. Previous studies reported that sepsis induces Leu resistance in skeletal muscles, which impairs its ability to prevent muscle wasting [14,15]. On the contrary, Leu supplementation was found to inhibit skeletal muscle degradation and enhance protein synthesis in lipopolysaccharide-challenged rats [16]. As far as we know, no study has investigated the role of Gln in sepsis-induced myopathies, and the anti-inflammatory and anti-oxidative properties of Gln may synergize with Leu to alleviate skeletal muscle damage. Therefore, we proposed to evaluate the effects of Gln and/or Leu administration on skeletal muscle injuries in sepsis. Cecal ligation and puncture (CLP) was used to induce murine sepsis in this study. This model has been extensively used to investigate sepsis-associated muscle wasting and the underlying mechanisms [17]. Antibiotics were also applied to imitate clinical treatment. We hypothesized that Gln and/or Leu administration would reduce oxidative stress, alleviate inflammatory responses, and decrease proteolytic pathways, which may attenuate sepsis-induced skeletal muscle injuries.

## 2. Results

### 2.1. Body Weight (BW) Changes and Gastrocnemius (GA) Muscle Weights

Compared to the sham control (C) group, mice in the septic control (S) group had lower BWs and GA muscle weights on day 4 postoperatively. There were no differences in BW loss among the sepsis groups, whereas Leu treatment alone (the L group) increased GA muscle weights on day 4 after CLP (Table 1).

### 2.2. Leukocyte Populations in the Blood

**Figure 1:**
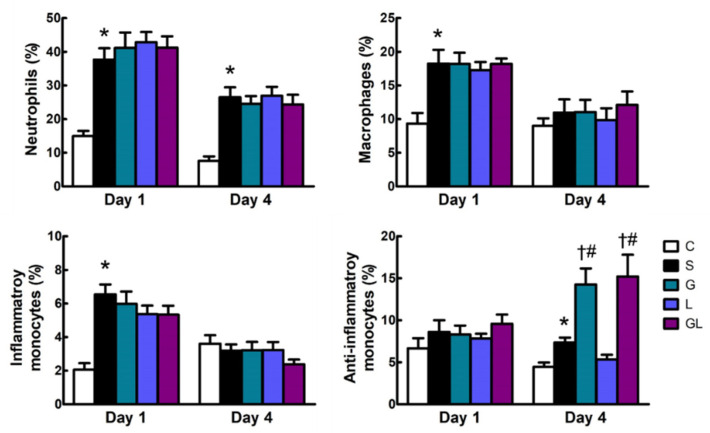
Blood leukocyte distribution. The groups are described in the legend to Table 1. Leukocyte populations are presented as the percentages of neutrophils (Ly6G^+^), macrophages (F4/80^+^), inflammatory monocytes (Ly6C^high^CCR2^+^), and anti-inflammatory monocytes (Ly6C^low^CX3CR1^+^) among blood leukocytes (CD45^+^). Student’s *t*-test was used to analyze differences between the C and S groups at the same time point. Differences among septic groups on the same day were analyzed by a one-way ANOVA with Tukey’s post-hoc test. * Significantly differs from the C group. † Significantly differs from the S group. # Significantly differs from the L group (*p* < 0.05).

The S group had higher neutrophil populations than the C group on both days 1 and 4 postoperatively. Additionally, percentages of macrophages and inflammatory monocytes were higher on day 1, while anti-inflammatory monocytes were higher on day 4 in the S group than those in the C group. Among the sepsis groups, mice treated with Gln (the G group) and Gln plus Leu (the GL group) had higher anti-inflammatory monocyte populations than the S and L groups on day 4 after CLP (Figure 1).

### 2.3. Leukocyte Populations in Muscles

**Figure 2:**
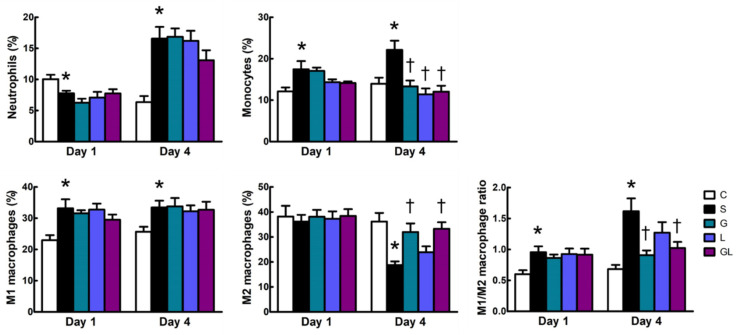
Leukocyte populations in muscles. The groups are described in the legend to Table 1. Leukocyte populations are presented as the percentages of neutrophils (Ly6G^+^), monocytes (Ly6C^high^), M1 macrophages (F4/80^+^CD206^-^), and M2 macrophages (F4/80^+^CD206^+^) among leukocytes (CD45^+^). The M1/M2 macrophage ratio was also demonstrated. Student’s *t*-test was used to analyze differences between the C and S groups at the same time point. Differences among septic groups on the same day were analyzed by a one-way ANOVA with Tukey’s post-hoc test. * Significantly differs from the C group. † Significantly differs from the S group (*p* < 0.05).

Lower neutrophils were exhibited on day 1, whereas the population was higher on day 4 after surgery in the S group than the C group. The S group had greater muscle monocyte infiltration than the C group on day 4 postoperatively. For macrophage populations, the S group had a higher level of the M1 subtype and the M1/M2 ratio on both days 1 and 4, while the M2 subtype was lower than the C group 4 days after surgery. Compared to the S group, amino acid-supplemented groups (G, L, and GL) had lower monocyte populations, whereas M2 macrophages and the M1/M2 ratio were higher in the G and GL groups on day 4 after CLP (Figure 2).

### 2.4. Calpain Activity and Malondialdehyde (MDA) Content in Muscle Tissues

**Figure 3:**
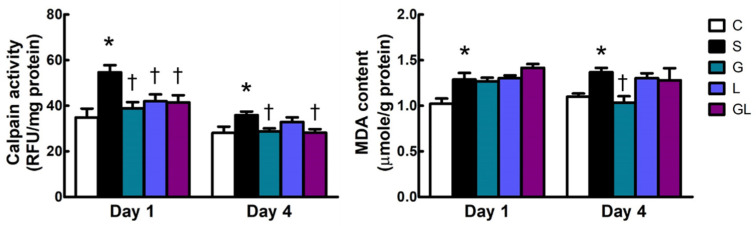
Calpain activity and malondialdehyde (MDA) content in muscles. The groups are described in the legend to Table 1. Student’s *t*-test was used to analyze differences between the C and S groups at the same time point. Differences among septic groups on the same day were analyzed by a one-way ANOVA with Tukey’s post-hoc test. * Significantly differs from the C group. † Significantly differs from the S group (*p* < 0.05).

The S group had higher calpain activity and MDA contents than the C group after surgery (Figure 3). Among the sepsis groups, all amino acid-supplemented groups had lower calpain activities than the S group on day 1, and the calpain inhibitory effect observed in the G and GL groups was prolonged to day 4 after CLP. There were no differences in MDA levels among all sepsis groups (Figure 3).

### 2.5. Muscle mRNA Expressions of Inflammatory and Mitochondrion-Related Genes

**Figure 4:**
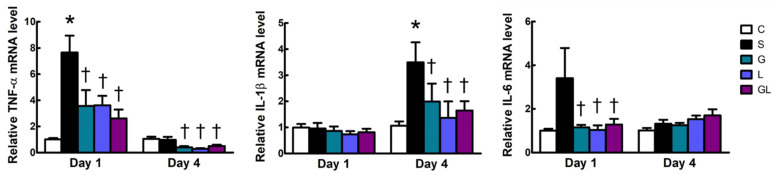
Muscle mRNA expressions of inflammatory genes. The groups are described in the legend to Table 1, and gene abbreviations are given in the footnotes to Table 2. Student’s *t*-test was used to analyze differences between the C and S groups at the same time point. Differences among septic groups on the same day were analyzed by a one-way ANOVA with Tukey’s post-hoc test. * Significantly differs from the C group. † Significantly differs from the S group (*p* < 0.05).

Compared to the C group, the S group had higher mRNA expressions of tumor necrosis factor (TNF)-α and interleukin (IL)-1β on days 1 and 4, respectively. The three amino acid-treated groups exhibited lower TNF-α, IL-1β, and IL-6 expression levels than those in the S group on day 1 and/or day 4 after CLP (Figure 4).

**Figure 5:**
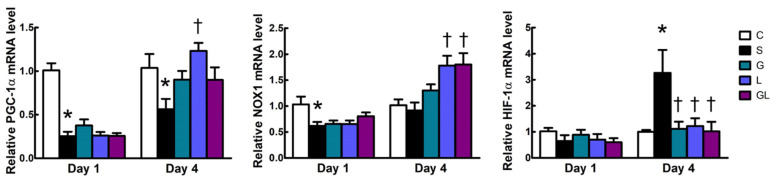
Muscle mRNA expressions of mitochondrion-related genes. The groups are described in the legend to Table 1, and gene abbreviations are given in the footnotes to Table 2. Student’s *t*-test was used to analyze differences between the C and S groups at the same time point. Differences among septic groups on the same day were analyzed by a one-way ANOVA with Tukey’s post-hoc test. * Significantly differs from the C group. † Significantly differs from the S group (*p* < 0.05).

The S group had lower mRNA expressions of peroxisome proliferative-activated receptor (PPAR)-γ coactivator (PGC)-1α, and NADPH oxidase 1 (NOX1) on day 1, whereas higher hypoxia-inducible factor (HIF)-1α expression on day 4 after surgery than the C group. Among all sepsis groups, the L group had higher PGC-1α expression, whereas the L and GL groups had higher expression levels of NOX1 on day 4 after CLP. All amino acid-treated groups showed lower HIF-1α gene expression than those in the S group 4 days post-CLP (Figure 5).

### 2.6. Muscle Histology

**Figure 6:**
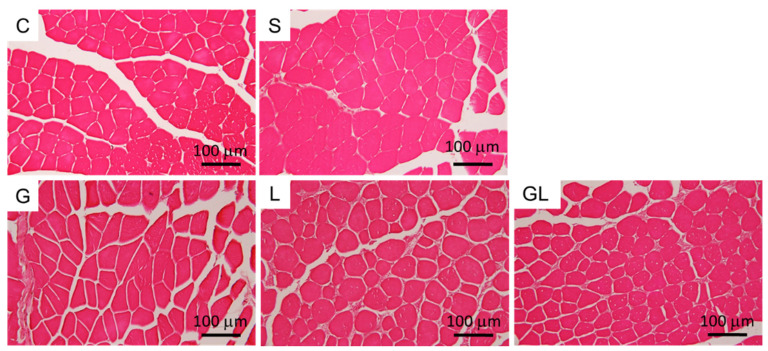
H&E staining of the gastrocnemius (GA) muscle. Representative histological images on day 4 postoperatively (200× magnification) are provided. The groups are described in the legend to Table 1.

GA muscle tissues of the C group exhibited uniform myofiber sizes with well-defined borders on day 4 postoperatively. Muscle disorganization and atrophy were not observed in septic mice, and amino acid treatment had no effect on GA muscle morphology on day 4 post-CLP (Figure 6).

## 3. Discussion

Intensive care unit (ICU)-acquired weakness is a major cause of long-term morbidity and mortality in critically ill patients, and sepsis survivors have higher risk of developing myopathies that worsen disease outcomes [6]. In this study, Gln and/or Leu were administered in a CLP-induced sepsis model to investigate the efficacy of specific amino acids in attenuating skeletal muscle damage. We found that Gln and Leu exhibited different protective effects on sepsis-induced muscle injuries. The findings showed that Gln prominently promoted an anti-inflammatory response, while Leu was more focused on enhancing the expression of mitochondrial function related genes. Both Gln and Leu provided beneficial effects of attenuating muscle protein degradation in sepsis.

Neutrophils and macrophages are activated during pathogenic infection as observed in sepsis. The infiltration of immune cells into tissues results in excessive inflammation and organ injury [18]. Circulating inflammatory monocytes are recruited to tissues, and subsequently differentiate into macrophages to provoke an inflammatory response and eliminate pathogens [19]. Macrophage activation is described as a dynamic process. During the onset of inflammation, tissue macrophages are mainly of the proinflammatory M1 type, whereas they may become M2 macrophages at a later stage which participate in resolving inflammation and promoting tissue repair [20]. In this study, we found that compared to the C group, percentages of blood neutrophils, macrophages, and inflammatory monocytes were elevated in the sepsis groups; meanwhile, neutrophil and monocyte infiltration and a higher M1/M2 macrophage ratio were observed in skeletal muscles of septic mice. Consistent with the polarized M1 phenotype, inflammatory cytokine mRNA expressions and MDA contents in muscles were also enhanced. MDA is a well-known end product of lipid peroxidation [21], which is used as an indicator of oxidative damage to cell membranes. These findings indicated more activation of immune cell infiltration that may aggravate inflammation and oxidative damage to muscle tissues.

In this study, several parameters associated with muscle protein degradation and mitochondrial function were analyzed. Calpains are Ca-activated cysteine proteases that play key roles in the disassembly of sarcomeric proteins and muscle protein breakdown. Calpain activation is critically involved in sepsis-induced muscle wasting [22]. Sepsis-induced mitochondrial alterations lead to inefficient muscle regeneration, which is also critical in sepsis-induced myopathies [23]. Several mitochondrion-related genes were assessed in the present study, including HIF-1α, NOX1, and PGC-1α. HIF-1α is induced by hypoxia and reactive oxygen species (ROS) [24]. HIF-1α regulates mitochondrial metabolism under hypoxic conditions to prevent excess mitochondrial ROS production [25]. NOX is a membrane-bound enzyme complex which catalyzes ROS formation. NOX1 is a member of the NOX family, which participates in cell growth [26] and can be triggered by mitochondrial ROS [27]. PGC-1α, a transcriptional coactivator, has powerful effects on suppressing ROS production, enhancing mitochondrial biogenesis, and preventing muscle wasting [28]. The findings of this study showed that concomitant with increased inflammatory cytokine expressions and lipid peroxidation, calpain activity and HIF-1α gene expression were also upregulated, whereas NOX1 and PGC-1α expression levels were downregulated in skeletal muscles of septic mice. These results suggest that sepsis activated the muscle-degradation process and impaired the mitochondrial redox status and biogenesis in skeletal muscles. The CLP sepsis model is able to induce GA and tibialis anterior (TA) muscle atrophy, which can be observed by hematoxylin and eosin (H&E) staining at days 4~5 after surgery [29,30]. However, the GA muscle morphology of septic mice was not altered in this study, which could be explained by the antibiotic intervention. As all septic mice survived in this study, we speculated that antibiotic treatment attenuated the severity of sepsis, which subsequently reduced sepsis-induced tissue injury. Morphological changes in skeletal muscles would be more obvious with a longer duration of sepsis.

In this study, several beneficial effects were observed when Gln and/or Leu were administered. First, Gln alleviated local and systemic inflammation, which may have attenuated sepsis-induced muscle protein breakdown and oxidative stress. Our findings showed that percentages of blood anti-inflammatory monocytes were elevated, whereas muscle monocyte infiltration and the M1/M2 macrophage ratio were reduced in the G and GL groups. Anti-inflammatory monocytes patrol blood vessel walls to remove cellular debris and participate in wound healing and the resolution of inflammation [31]. The polarization of macrophages shifting from the M1 toward the M2 type in muscles may promote skeletal muscle repair [32,33]. Consistent with those findings, TNF-α, IL-1β, and IL-6 mRNA expressions in muscles were downregulated. A study performed by our laboratory also revealed that Gln supplementation reduced the muscle M1/M2 ratio and accelerated muscle regeneration in diabetic mice with limb ischemia [11]. Inflammation is linked to oxidative stress [34] and tissue hypoxia [35]. The anti-inflammatory microenvironment in muscles may have been partly responsible for the reductions in lipid peroxidation and HIF-1α expression observed in the Gln-treated group. The anti-inflammation and lower calpain activities exerted by Gln administration suggest that damage and degradation of muscles were attenuated. Second, Leu has benefits in reducing inflammation, suppressing proteolysis, and enhancing the expression of mitochondrial function-related genes in skeletal muscles of septic mice. In this study, Leu was found to suppress monocyte infiltration, calpain activity, and mRNA expressions of inflammatory cytokines and HIF-1α, suggesting that Leu administration may attenuate muscle wasting in sepsis by its anti-inflammatory actions. We noted that calpain suppression had vanished, and NOX1 mRNA expression was enhanced on day 4 postoperatively in Leu-treated mice. Although NOX1 participates in ROS formation, upregulation of NOX1 by Leu did not aggravate lipid peroxidation in the present study. The effects of ROS on muscle regeneration are bifacial in the context of mitochondria [36]. High levels of ROS may lead to mitochondrial dysfunction, whereas moderate levels trigger mitochondrial biogenesis by PGC-1α upregulation in skeletal muscles [37]. A previous study showed that Leu enhances the oxidative capacity and increases PGC-1α expression and mitochondrial density in skeletal muscle cells [38]. Exogenous Leu administration was found to increase muscle protein synthesis under in vivo and in vitro conditions [39,40,41,42]. In this study, PGC-1α expression was enhanced by Leu, suggesting that Leu administration may be conducive to muscle regeneration in sepsis. However, it was reported that CLP-induced sepsis impairs the ability of Leu to stimulate muscle protein synthesis [14]. The beneficial effects of Leu on muscle repair observed in this study might have been partially assisted by antibiotic treatment. Further studies are needed to verify our speculation. In this study, we found that there were some different modulatory effects between Gln and Leu. However, Gln combined with Leu did not show a greater extent of changes than those exhibited by Gln or Leu alone; thus, there were no synergistic effects when Gln and Leu were simultaneously administered.

**Figure 7:**
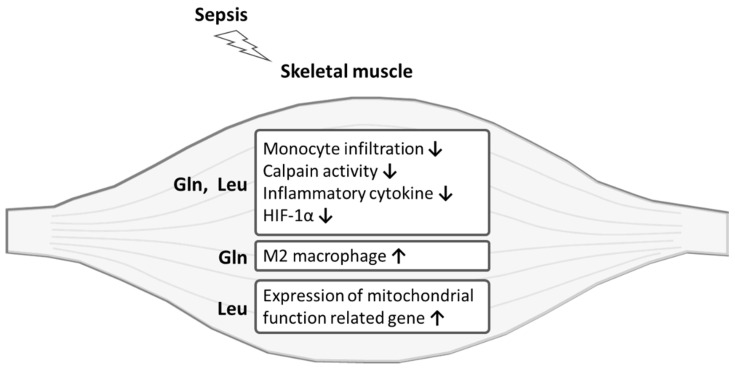
Schematic overview of the protective effects l-glutamine (Gln) and l-leucine (Leu) on sepsis-induced skeletal muscle injuries. HIF, hypoxia-inducible factor.

In summary, this study showed for the first time that Gln and Leu administration had favorable effects on muscle damage in sepsis (Figure 7). Both Gln and Leu suppressed monocyte infiltration into muscles, attenuated inflammatory mediator production, inhibited calpain activity, and downregulated HIF-1α mRNA expression in the early and/or late phases of sepsis. Gln had a greater capacity to promote an anti-inflammatory response by polarizing M1 macrophages toward the M2 subtype, while the beneficial effects of Leu treatment focused on suppressing muscle protein breakdown and enhancing the expression of mitochondrial function related genes. However, Gln administration failed to show synergism with Leu against sepsis-induced muscle injuries.

## 4. Materials and Methods

### 4.1. Animals

Eight-week-old male C57BL/6J mice were used in this study. Mice were conventionally housed in a temperature- and humidity-controlled room with a 12 h light/dark cycle and were given free access to standard chow diet (Rodent Laboratory Chow no. 5001, Ralston Purina, St. Louis, MO, USA) and drinking water ad libitum. Care of laboratory animals was in full compliance with the Guide for the Care and Use of Laboratory Animals (National Research Council, 1996) and protocols were approved by the institutional Animal Care and Use Committee of Taipei Medical University (LAC-2020-0182).

### 4.2. Experimental Protocols

After 1 week of acclimation, mice were randomly divided into one sham control group (the C group; *n* = 12), one septic control group (the S group; *n* = 20), and three septic groups with an amino acid intervention (the G, L, and GL groups; *n* = 20 in each group). Mice in the septic groups were subjected to CLP to induce polymicrobial sepsis as previously described [43]. Briefly, mice were anesthetized with an intraperitoneal (i.p.) injection of zoletil (Virbac, Carros, France; 40 mg/kg BW) and rompun (Bayer, Leverkusen, Germany; 10 mg/kg BW). After making a 1.5 cm abdominal incision, the cecum was fully exposed and tightly ligated at 50% of the cecum length with 3-0 silk sutures, and then punched through once with a 22-gauge needle. A small amount of cecum contents was gently squeezed out and smeared onto the surface of the cecum before replacing it into the abdomen. The abdominal musculature and skin incisions were separately sutured using 3-0 silk sutures, and bupivacaine (Marcaine, AstraZeneca, Monts, France; 8 mg/kg BW) was locally applied for analgesia at the incision site before closing the skin. Mice in the C group underwent a sham operation, for which an identical laparotomy was performed but the cecum was neither tied nor punctured. All mice were allowed free access to food and water after surgery.

Antibiotic treatments and amino acid interventions began at 1 h after the operation, and animals were re-injected every 24 h via the i.p. route. Both sham and septic mice were treated with the antibiotic Ertapenem (INVANZ, Merck, Whitehouse Station, NJ, USA; 75 mg/kg BW). For the amino acid intervention, septic mice were injected with Gln (0.5 g/kg BW) in the G group, Leu (0.1 g/kg BW) in the L group, and Gln (0.5 g/kg BW) plus Leu (0.1 g/kg BW) in the GL group. Mice in the C and S groups received identical volumes of saline. A commercial l-alanyl-l-glutamine (Ala-Gln) solution (Dipeptiven, Fresenius Kabi, Homburg, Germany) was used for Gln treatment at a dose of 0.75 g Ala-Gln/kg BW (equivalent to 0.5 g Gln/kg BW), and Leu was purchased from Sigma-Aldrich (St. Louis, MO, USA). Compared to the S group, amino acid administration provided ~0.3% extra energy from the Ala-Gln solution and ~0.04% extra energy from the Leu infusion. This amount of Gln exhibited immunomodulatory effects in septic mice [44,45], and antiproteolytic properties in mice with dystrophic muscles [46]. The dosage of Leu was selected based on a previous study which showed the ability to activate protein synthesis signaling in mouse skeletal muscle [47].

On day 1 after the operation, half of the mice in each group were anesthetized and killed by cardiac puncture, and the rest were killed on day 4 post-operation. Blood was collected in heparin-containing tubes, and the GA and TA muscles were dissected from both legs. Blood and the TA muscle of the right leg were used fresh for a flow cytometric analysis. The GA muscle of the right leg was fixed with 4% paraformaldehyde in phosphate-buffered saline (PBS) for a histological examination. The remaining muscle tissues were snap frozen in liquid nitrogen and kept at −80 °C until being processed for further analysis.

### 4.3. Distribution of Circulating Leukocytes

To analyze the leukocyte population in the blood, 100 μL aliquots of whole blood were incubated with antibodies against mouse leukocyte surface antigens at 4 °C in the dark for 30 min. The antibodies included peridinin-chlorophyll (PerCP)-conjugated anti-mouse CD45 (Biolegend, San Diego, CA, USA), fluorescein isothiocyanate (FITC)-conjugated anti-mouse Ly6G (Biolegend), Pacific blue-conjugated anti-mouse F4/80 (Biolegend), phycoerythrin (PE)-conjugated anti-mouse Ly6C (eBioscience, San Diego, CA, USA), allophycocyanin (APC)-conjugated anti-mouse C–C chemokine receptor type 2 (CCR2) (R&D Systems, Minneapolis, MN, USA), and Alexa Fluor^®^ 700-conjugated anti-mouse C-X3-C motif chemokine receptor 1 (CX3CR1) (Biolegend).

After red blood cells were lysed, stained cells were suspended in staining buffer (PBS with 0.5% bovine serum albumin) and analyzed with an Attune NxT flow cytometer (ThermoFisher Scientific, Waltham, MA, USA). CD45-positive (CD45^+^) cells were gated and determined to be leukocytes. Leukocyte populations are presented as percentages of neutrophils (Ly6G^+^), macrophages (F4/80^+^), inflammatory monocytes (Ly6C^hig^CCR2^+^), and anti-inflammatory monocytes (Ly6C^low^CX3CR1^+^) among leukocytes (CD45^+^).

### 4.4. Leukocyte Populations in Muscles

TA muscles were minced and digested in Dulbecco’s modified Eagle medium with 0.1% type II collagenase (Thermo Fisher Scientific) at 37 °C for 1 h. Digested tissues were pressed and filtered through a 70 µm strainer (Becton Dickinson, Franklin Lakes, NJ, USA) with a syringe plunger. Cell pellets obtained by centrifugation were re-suspended in staining buffer. Cell suspensions were pre-incubated with an anti-mouse CD16/CD32 antibody (Biolegend) on ice for 5 min to block Fcγ receptors, and then stained with PerCP-conjugated anti-CD45, FITC-conjugated anti-Ly6G, Pacific blue-conjugated anti-F4/80, PE-conjugated anti-Ly6C, and APC-conjugated anti-CD206 antibodies at 4 °C in the dark for 30 min. Stained cells were run through a flow cytometer (Attune NxT, ThermoFisher Scientific). Leukocyte populations in muscles are presented as percentages of neutrophils (Ly6G^+^), monocytes (Ly6C^high^), M1 macrophages (F4/80^+^CD206^-^), and M2 macrophages (F4/80^+^CD206^+^) among muscle leukocytes (CD45^+^).

### 4.5. Calpain Activity and MDA Concentration

Calpain activities and MDA contents in the TA muscle were determined using commercial fluorometric assay kits (Calpain Activity Assay Kit, Abcam, Cambridge, UK; TBARS Assay Kit, Cayman Chemical, Ann Arbor, MI, USA). Tissue homogenization and assay procedures were performed according to the manufacturer’s instructions. Protein concentrations of homogenized samples were measured with a BioRad Bradford protein assay (Hercules, CA, USA). The measured calpain activity and MDA level were expressed relative to the total protein content of tissue homogenates.

### 4.6. Real-Time Polymerase Chain Reaction (PCR)

In order to obtain total RNA, GA muscles were homogenized in Trizol reagent (ThermoFisher Scientific), followed by chloroform extraction and isopropanol precipitation. RNA was reverse-transcribed into complementary (c)DNA using oligo (dT) 18 primers with a commercial cDNA synthesis kit (ThermoFisher Scientific). For analysis of gene expression, 50~100 ng of cDNA was amplified in a 25 μL reaction containing 50~200 nM of each primer and 1× QuantiNova SYBR green master mix reagent (Qiagen, Hilden, Germany). Amplification was performed using a Rotor-Gene Q real-time cycler (Qiagen) under the following conditions: 50 °C for 2 min and 95 °C for 10 min, followed by 40 cycles of 95 °C for 15 s and 60 °C for 1 min, with a final dissociation curve analysis. Primers used in this study are listed Table 2. All samples were analyzed in triplicate. Target gene expressions were normalized to hypoxanthine-guanine phosphoribosyltransferase (HPRT) expression as an endogenous control, and relative gene expression levels were calculated by the equation 2^−ΔΔCt^ (ΔCt indicates the difference in threshold cycles between the test gene and HPRT, and ΔΔCt indicates the difference of ΔCt between the C and septic groups).

### 4.7. Histology

Paraformaldehyde-fixed GA muscles were embedded in paraffin and sliced into 5-μm-thick sections, followed by H&E staining to examine the morphology of muscle tissues. Five fields per section were digitally captured at 200× magnification using an Olympus BX43 Upright microscope (Waltham, MA, USA) and a Canon EOS 700D digital camera (Tokyo, Japan).

### 4.8. Statistical Analysis

Data are presented as the mean ± standard error of the mean (SEM). Statistical analyses were performed with GraphPad Prism 5 software (GraphPad Software, San Diego, CA, USA). Student’s *t*-test was used for comparing values between the C and S groups, and differences among the four septic groups were determined using a one-way analysis of variance (ANOVA) followed by Tukey’s post-hoc test. Differences between the groups were considered statistically significant at *p* < 0.05.

## 5. Conclusions

Both Gln and Leu had prominent abilities to attenuate inflammation and degradation of skeletal muscles in the early and/or late phases of sepsis. Moreover, Gln promoted the switch of leukocytes toward an anti-inflammatory phenotype, while Leu treatment maintained bioenergetics function in the skeletal muscle.

## Figures and Tables

**Table 1:** Body weight (BW) changes and gastrocnemius (GA) muscle weights.

	C	S	G	L	GL
BW change (%)	−2.73 ± 0.19	−15.61 ± 0.95 *	−15.19 ± 0.95	−14.05 ± 0.99	−15.35 ± 0.88
GA muscle weight (% of BW)	1.18 ± 0.01	1.06 ± 0.03 *	1.12 ± 0.02	1.16 ± 0.02 †	1.14 ± 0.02

C, sham control group; S, septic control group; G, the septic group with l-glutamine intervention; L, the septic group with l-leucine intervention; GL, the septic group with l-glutamine and l-leucine intervention. Data are presented as the mean ± SEM. Student’s *t*-test was used to analyze differences between the C and S groups. Differences among the septic groups were analyzed by a one-way ANOVA with Tukey’s post-hoc test. * Significantly differs from the C group. † Significantly differs from the S group (*p* < 0.05).

**Table 2:** Primer sequences for the real-time polymerase chain reaction.

Gene	Primer Sequences (5′ to 3′)	Accession Number
TNF-α	F: CCCTCACACTCAGATCATCTTCT R: GCTACGACGTGGGCTACAG	NM_013693.3
IL-1β	F: TGCCACCTTTTGACAGTGATG R: ATGTGCTGCTGCGAGATTT	NM_008361.4
IL-6	F: GGGACTGATGCTGGTGACAA R: ACAGGTCTGTTGGGAGTGGT	NM_001314054.1
PGC-1α	F: CTGCGGGATGATGGAGACAG R: TCGTTCGACCTGCGTAAAGT	NM_008904.2
NOX1	F: GGAGTGGCATCCCTTCACTC R: GGCATTGGTGAGTGCTGTTG	NM_172203.2
HIF-1α	F: CTGTTATGAGGCTCACCATCAG R: CAGTCCATCTGTGCCTTCATC	NM_001313919.1
HPRT	F: AGCCTAAGATGAGCGCAAGT R: TTACTAGGCAGATGGCCACA	NM_013556.2

TNF, tumor necrosis factor; IL, interleukin; PGC, peroxisome proliferative-activated receptor-γ coactivator; NOX1, NADPH oxidase 1; HIF, hypoxia-inducible factor; HPRT, hypoxanthine-guanine phosphoribosyltransferase; F, forward; R, reverse.
